# Molecular Epidemiology of *Aspergillus fumigatus* in Chronic Pulmonary Aspergillosis Patients

**DOI:** 10.3390/jof7020152

**Published:** 2021-02-20

**Authors:** Mireille H. van der Torre, Hongwei Shen, Riina Rautemaa-Richardson, Malcolm D. Richardson, Lilyann Novak-Frazer

**Affiliations:** 1 Mycology Reference Centre Manchester, ECMM Centre of Excellence in Clinical and Laboratory Mycology and Clinical Studies, Manchester University NHS Foundation Trust, Wythenshawe Hospital, Manchester M23 9LT, UK; mireille.vandertorre-2@manchester.ac.uk (M.H.v.d.T.); Riina.Richardson@manchester.ac.uk (R.R.-R.); Malcolm.Richardson@manchester.ac.uk (M.D.R.); 2 Division of Infection, Inflammation and Respiratory Medicine, Faculty of Biology, Medicine and Health, University of Manchester, Manchester M13 9PL, UK; hongweishen@smu.edu.cn; 3 Department of Infectious Diseases, Manchester University NHS Foundation Trust, Wythenshawe Hospital, Manchester M23 9LT, UK

**Keywords:** *Aspergillus fumigatus*, molecular epidemiology, chronic pulmonary aspergillosis, genotyping, genetic diversity

## Abstract

Molecular fungal genotyping techniques developed and employed for epidemiological studies have understandably concentrated on establishing the genetic diversity of *Aspergillus fumigatus* in invasive aspergillosis due to its severity, the urgency for treatment, and the need to demonstrate possible sources. Some early studies suggested that these strains were phenotypically, if not genotypically, different from others. However, with improved discrimination and evaluations, incorporating environmental as well as clinical isolates from other *Aspergillus* conditions (e.g., chronic pulmonary aspergillosis and cystic fibrosis), this premise is no longer upheld. Moreover, with the onset of increased global triazole resistance, there has been a concerted effort to incorporate resistance profiling into genotyping studies and the realisation that the wider population of non-immunocompromised aspergillosis patients are at risk. This review summarises the developments in molecular genotyping studies that incorporate resistance profiling with attention to chronic pulmonary aspergillosis and an example of our UK experience.

## 1. Introduction

Chronic pulmonary aspergillosis (CPA) is a widely diverse disease, spanning from relatively non-progressive conditions, such as aspergilloma, to severe and ultimately fatal conditions, such as chronic necrotising pulmonary aspergillosis (CNPA), now called subacute invasive pulmonary aspergillosis (SAPA) [1]. CPA can occur in patients with underlying pulmonary disease [2] and presents with persistent cough, haemoptysis, weight loss, breathlessness, and fatigue, amongst other symptoms, and may lead to respiratory failure through progressive fibrosis and lung destruction [1]. Furthermore, growing evidence suggests that the global increase in resistance to antifungal triazole drugs, the mainstay of treatment for chronic conditions [3], is likely to imperil therapeutic options [4]. However, few true epidemiological studies have been conducted that could help our understanding of the course and evolution of this chronic disease in terms of pathogen–host relationships. Understandably, the current knowledge base is mainly from genotyping studies of invasive pulmonary aspergillosis (IPA) cases, although there are encouraging signs that the chronic conditions are being addressed with the onset of improved typing technologies.

Molecular epidemiology is a branch of epidemiology that focuses on the genetic background of the pathogens and their relationship among hosts [5]. The main goal of molecular epidemiology is to understand pathogen–host relationships and identify the risk factors of the disease (transmission, disease manifestation and disease progression), which can help in the development of more effective prevention and control strategies at individual and population levels. As such, a taxonomic study of the infectious organism on its own is not epidemiology, although the same methods can be used [5]. Questions that can be asked are as follows: Are certain fungal genotypes more likely to occur in specific patient groups or to develop into specific forms of aspergillosis? Are there different genotypes (and how many?), and if so, are they randomly distributed or do they cluster geographically? Is there a common source in an outbreak?

Fungal genotyping is a tool used in epidemiological studies and is defined as genetic analysis of clinical and/or environmental isolates that is performed to assign strain-specific (i.e., below species level) fingerprints. Genotyping should generate molecular markers of sufficient discriminative power to enable comparisons among strains and study their genetic relatedness. Previously, genotyping techniques have been employed to study the population dynamics and transmissibility of *Aspergillus* species in several clinical and environmental settings, usually with a focus on IPA and nosocomial outbreaks (for examples, see [6,7,8,9,10,11,12,13]). Using these data, environmental source(s) of infection can be determined. In contrast, multiple isolations of an organism may occur in a single patient over a particular time frame; if sufficiently discriminative, genotyping may enable further understanding of how a fungal pathogen behaves in a particular host. Furthermore, a particular genotype may be associated with antifungal resistance, for example, in patients with CPA and other chronic *Aspergillus* conditions. One reason for the paucity of genotyping studies of CPA patients is that until the onset of global patterns of pan-triazole resistance, the origin of resistant strain development was thought to be mainly due to persistence of *A. fumigatus* within the patient lung [14]. Although this may still be the case for most patients, the possibility of infection by environmental strains must now be tracked through epidemiological studies to deliver improved outcomes for CPA patients.

In this review, we provide an update on the latest *A. fumigatus* genotyping studies with a focus on research performed in CPA patient groups and environmental isolates. We also summarise the evidence from a small study that represents a snapshot of the resistance genotypes and geographic distribution among CPA patients in the UK served by the National Aspergillosis Centre (NAC) in Manchester.

## 2. Epidemiology of *Aspergillus fumigatus*

The main fungal pathogen associated with CPA is *Aspergillus fumigatus*. It is a fast-growing saprobe, commonly found on decaying vegetation, but it has also adapted the enzymatic machinery to become a facultative human pathogen [15,16]. This species is found ubiquitously in the environment in soil, water, and air, which also includes patients’ homes and hospital facilities [6,17,18]. Worryingly, triazole-resistant strains are also found in the environment, domiciles, and hospitals worldwide [7,8,9]. For example, at the University Hospital of Besançon (UHB), flower beds harbouring triazole-resistant *Aspergillus fumigatus* (AR*Af*) next to the hospital site were thought to be the source of AR*Af* isolated from hospital corridors and clinical patient samples; however, comparative genotype analysis of these species is needed to confirm this hypothesis [10].

In considering the epidemiology of CPA, it is useful to first look into the global burden of this disease. Approximately three million people worldwide suffer from CPA and the global burden is thought to be significantly higher in Asia compared to other continents [19]. It is estimated that in 2019, 1.2 million patients developed CPA after pulmonary tuberculosis (PTB), over 410,000 patients progressed to CPA as a complication of allergic bronchopulmonary aspergillosis (ABPA), and almost 72,000 patients progressed to CPA as a complication of pulmonary sarcoidosis [20,21,22]. Moreover, global triazole resistance rates are variable, from 3.2 to 26.1% among the 21 centres involved in a recent global surveillance study [23].

Given the widespread existence of *A. fumigatus*, its association with human diseases, and the compounding factor of emergence of drug-resistant strains, it is important to investigate the transmission and virulence of *A. fumigatus* by means of molecular epidemiological studies. Genotyping the causative agent can provide useful information about the disease including its evolution, geographical distribution, route of transmission, source, host risk factors, and strain-specific virulence. In one of the earliest typing studies (with samples from IPA patients), Tang and colleagues hypothesised that certain isolates were more pathogenic than others [11]. In their study, diverse environmental isolates were found in the hospital during building works but none of those genotypes were detected in patient samples, which were unique. This premise seemed to be supported by a later study, which found that virulence was strain-dependent and correlated with the nature of infection (IPA vs. non-IPA) and the host’s immune status [24], although the genotypic distinction between invasive and non-invasive settings was not complete. In a later study, the same group showed that isolates from lung transplant patients consisted of numerous genotypes but that the isolates from three IPA patients demonstrated a unique genotype [25], thus supporting the Tang study. However, after further work by other groups, two early reviews determined that these genetic analyses did not have the resolving power to discriminate between clinical and environmental isolates, concluding that any environmental strain was a potential pathogen if it encountered an appropriate host [26,27]. In the last 30 years, the development and implementation of new typing methods with higher discriminatory indices have provided considerable clarity to the research in this field.

## 3. *Aspergillus fumigatus* Genotyping Methods

Molecular typing, consisting of methods for the direct examination of DNA sequences, was developed to supersede phenotypic methods, considered to be less reliable and discriminative [28]. In addition to their primary use for discriminating members of the species *A. fumigatus*, genotyping studies have led to the discovery of new pathogenic species, e.g., *A. lentulus* [29], and to the discovery of several cryptic *Aspergillus* species not known previously to be pathogenic (reviewed in [26]). Two early reviews discussed thoroughly the benefits and disadvantages of the early techniques, including multilocus sequence typing (MLST) and a method to detect microsatellite length polymorphisms (MLPs) [26,27]. The latter technique consists of a panel of nine markers, each comprising numerous short tandem repeats (STR) (or variable number of tandem repeats, VNTR, in some papers) for fingerprinting *A. fumigatus* isolates (or STRA*f*) [30]. The STRA*f* technique achieves a high discriminatory power and remains the current gold standard [31]. As STR polymorphisms are based on varying numbers of repeated short DNA fragments in non-coding regions, they are expected to be more polymorphic and rapidly evolving than, for example, MLST markers, which are based on discriminating single-nucleotide polymorphisms (SNPs) in housekeeping genes [32]. This was confirmed in an early comparison of the clinical isolates from twelve patients with IPA [33]. However, both methods are useful depending on the purpose of the investigation. Varga’s review [26] also summarised the outcomes of performing these typing assays, thus providing a comprehensive analysis of the epidemiology (environmental sources, population structure, and role of *MAT* loci on strain evolution) of *A. fumigatus* infections at that time. During this period of intense genotyping method improvements, a simpler technique, based on the variable repeats within a cell surface protein [34], was also developed that had an intermediate discriminatory power compared to MLST and STRA*f* [32]. Klaassen and Osheriv [31] reviewed two further technologies, coding tandem repeats [35] and retrotransposon insertion-site context (RISC) typing [36], adding to the diversifying list of genotyping technologies. The different molecular typing techniques were evaluated in terms of applicability, discriminatory power, and technical and interpretative capabilities [37], including in the most recent review [32]. These comprehensive reviews recommended genotyping techniques based on the epidemiological question, demonstrating that discriminatory power was key due to the high genetic variability of *A. fumigatus*. Despite the diverse typing technologies, the development of a gold standard approach, and burgeoning interest for epidemiological studies, the paucity of investigations of *A. fumigatus* isolates from CPA patients was clear in the reviews of the time [26,27,31,37].

Subsequent developments of the gold standard microsatellite-based typing assays concentrated on improving their interlaboratory reproducibility and interpretation by introducing ladders [38,39]. A further improvement was to multiplex the PCR component of the STRA*f* technique while reducing the number of short tandem repeat polymorphism microsatellite markers from nine to eight by using a revised primer set (selecting preferentially trinucleotide, tetranucleotide, and pentanucleotide repeats), thus refining the discriminatory power to 0.9997 [40]. Although the technical difficulties of the original STRA*f* PCRs were discussed comprehensively [32] and seemed to be alleviated [40], this latter eight-marker methodology, designated nSTRA*f*, does not seem to have been readily adopted. The stability of the original STRA*f* markers was also investigated recently: the trinucleotide repeats 3A and 3C were found to alter by one tandem repeat unit in three of five *A. fumigatus* strains when clonally expanded more than 5–36 generations, suggesting there was a very low level of instability that needed to be accounted for in genotyping investigations using this method [41]. Notwithstanding these considerations, microsatellite profiling has been taken up broadly despite its requirement for specialised equipment and technical expertise. Recent typing studies have not only revealed the existence of two genetically isolated groups within global *A. fumigatus* populations but also provided evidence of recombination [42]. Moreover, a recent web-based programme, called AfumID, provides an effective tool for assessing the placement of strains within a global population of resistant strains typed with the STRA*f* method [43].

To provide a genotyping method that could be integrated into any clinical laboratory undertaking routine Sanger sequencing, a new method was devised, based on hypervariable tandem repeats within the exons of three surface protein-coding genes (TRESP) conserved among filamentous fungi [44]. This method was developed by enhancing the original, rapid single-locus sequence typing method based on coding tandem repeats in the putative hypervariable cell surface protein A (*cspA*) (CSP typing) [45] that was used previously to discriminate clinical outbreak isolates from across North America [35]. The two additional genes included an MP-2 antigenic galactomannan protein (MP2) and a hypothetical protein with a CFEM domain (CFEM) [44]. Later, to improve the discriminative index to D = 0.9972, a fourth gene, *erg4B*, was added and the method was renamed TRESPERG [46]. Subsequent analyses have used either of the microsatellite typing STRA*f*, hypervariable CSP typing, or MLST typing methods in combination with resistance profiling to improve our understanding of the population genetics of *A. fumigatus* [43,46,47,48,49,50,51,52,53,54,55,56,57,58,59,60,61], although not all these reports include isolates derived from CPA patients.

Genome sequencing methods, particularly those that combine sequence differentiation with triazole susceptibility information, will provide the ultimate magnifying glass for genotypic evaluation, although few to date relate information in the context of CPA. A recent study using next-generation sequencing (NGS) to identify sequential mutations in *A. fumigatus* arising during infections in two patients, one with IPA and the other with an aspergilloma, demonstrated unique genotypes between the patients but identical microsatellite types among the sequential isolates from each patient. However, NGS allowed distinction among all the strains from each patient and showed numerous nonsynonymous mutations in successive isolates from both patients and clearly different phenotypes and resistance patterns, confirming the superiority of discrimination with this technique. The most interesting observation in the isolates from the chronic aspergillosis patient was that large genomic deletions were apparent, not present in the IPA patient, suggesting these deletions may provide an evolutionary advantage to isolates in chronic conditions [62]. Abdolrasouli et al. [63] used whole-genome sequencing (WGS), but not other genotyping methods for comparison, to carry out high-resolution SNP analysis of 24 *A. fumigatus* isolates, collected worldwide from clinical and environmental sources, including triazole-resistant and susceptible strains. They found that, apart from a single clade in Indian isolates, clinical and environmental isolates were genetically indistinguishable. Moreover, their analysis showed that triazole susceptibility/resistance, including environmental pan-triazole resistance, occurred in diverse genetic backgrounds [63]. Interestingly, this work also confirmed the previous observation that large deletions were apparent in some genomes, although whether these were present in clinical isolates from CPA patients could not be determined.

The most recent study using microsatellite and WGS typing of isolates from a patient suffering with X-linked chronic granulomatous disease, severe chronic obstructive pulmonary disease (COPD), and ABPA, who over the course of their treatment developed an aspergilloma, revealed 248 nonsynonymous SNPs [64,65]. Microsatellite typing determined that all isolates were isogenic, although independent analysis of the subset of STRA*f* markers using AfumID [43] elucidated different genotypes in the early strains, even when the instability of markers 3A and 3C was considered. Clearly, NGS/WGS sequencing techniques are superior to other genotyping methods, although combining markers, such as STRA*f* and TRESPERG, may approach the discriminatory power when the latter approach is not possible, particularly if STRA*f* and/or TRESPERG results suggest strain differences. Previous reviews have summarised eloquently the principles and characteristics of early typing techniques, such as random amplified polymorphic DNA (RAPD), multilocus enzyme electrophoresis (MLEE), and methods based on restriction endonuclease-generated DNA fragment patterns [26,27,32,33,37]. When combined to improve discrimination sufficiently, these typing methods sustain an important role in research but may not be practicable in a clinical setting. The advantages and disadvantages of the more recent *A. fumigatus* genotyping approaches discussed here are summarised in Table 1.

## 4. *Aspergillus fumigatus* Genotyping Study Results

Genotyping studies contribute to our knowledge of the genetic background of *A. fumigatus* and its population dynamics in various clinical as well as environmental settings and may help to improve clinical outcomes. Previous genotyping studies of *A. fumigatus* from IPA patients have been reviewed extensively [26,27,32]. In this review, we provide an update on the latest genotyping studies with a focus on research performed in CPA patient groups, where possible, and on environmental isolates.

### 4.1. Distinguishing the Nature of Infection

Part of molecular epidemiology concerns using genotyping techniques to ascertain the pathogen-related factors that influence disease manifestation [5]. Past studies investigated whether *A. fumigatus* isolated from IPA patients was distinct from other strains (reviewed in [26,27]). A significant difficulty in drawing conclusions from these early studies is that many predate the classifications that would now be used to stratify IPA patients according to the revised criteria of the European Organisation for Research and Treatment of Cancer (EORTC) [67]. A study of the local epidemiology of *A. fumigatus* analysed from 52 airway specimens from 12 IPA patients in three centres revealed that only one patient in each centre demonstrated a single genotype, and that the rest of the patients had mixed genotypes that were unique to them and unique among the three centres [33]. This study attested to the diversity of isolates found using the STRA*f* method and confirmed previous findings [11,25]. In a later study, using the precursor method to STRA*f* [68], isolates from IPA patients were found to be less variable than and different to those from patients colonised with *A. fumigatus*, although there was some crossover in patients with mixed genotypes [69]. In contrast, a more recent study of 1373 isolates from 95 patients that used the 2008 EORTC criteria to stratify IPA patients from those colonised with *A. fumigatus* revealed that although STRA*f* detected 395 genotypes, it could not discriminate between isolates from IPA patients and those who were colonised [70]. Moreover, in the previously reviewed study that suggested RAPD typing could differentiate among *A. fumigatus* isolates derived from patients with IPA and lung transplant patients with a variety of other aspergillosis conditions [25], the stratification of the patients included in the study was uncertain and the discriminatory power was not ideal [26].

One of the few genotyping studies performed on *A. fumigatus* strains from IPA and CPA patients revealed the existence of 99,088 SNPs using NGS [66] and illustrates the importance of diagnostic classification [3]. The genotypes from 17 Japanese strains were analysed from patients with either chronic necrotising pulmonary aspergillosis (now called subacute invasive pulmonary aspergillosis, or SAPA), defined by the authors as IPA in their previous study [62], or pulmonary aspergillomas, the most benign form of CPA [1]. The STRA*f* genotypes of the set of isolates from both patients were unique but isogenic. However, the three clades that resulted from NGS analysis did not correlate solely with either pathological condition nor were any SNPs found to be associated with the medication history profile [66]. Interestingly, if the isolates from the SAPA patient had instead been classified as the extreme end of the chronic aspergillosis spectrum [1,62], the fact that isolates from either patient did not segregate into different clades would not have been so surprising. This study did illustrate that NGS had far superior discrimination as the novel SNPs offer prospects for further investigation of the nature of infection caused by *A. fumigatus* and the mechanisms used to adapt to the host, although there is little support for differentiation among isolates causing invasive and chronic diseases.

Until further studies include the use of higher-resolution genotyping in conjunction with investigations that elucidate the adaptability of *A. fumigatus* to generate genetic diversity [14], the genetic differences that predispose some strains of *A. fumigatus* to cause IPA, simply colonise, or eventually cause chronic infections remain unknown. However, *A. fumigatus* is only one factor in this pathogen–host relationship. The breadth of the host spectrum between allergic and invasive aspergillosis, the former characterised by immune hyperactivity and the latter by immune dysfunction, with the chronic conditions somewhere within this spectrum [2], suggests the infective organisms should differ. Recent studies have shown that *A. fumigatus* virulence is dependent on the ability to adapt to stresses in the lung, such as hypoxia, nutrient availability, and engulfment by host phagocytes [71], which suggests that although any strain may be able to colonise, it is those strains that adapt to the lung environment that cause infection, presumably quickly in the case of IPA and over time in the case of CPA. Until further comparisons dissect the pathogen and host at the genomic level and are complemented by phenotypic/metabolomic analyses, this will remain an intriguing question.

### 4.2. Genomic Dynamics during Infection

Genotypic analyses of multiple isolates obtained from single patients over time demonstrate the adaptability of *A. fumigatus* to the host environment during infection, which is especially pertinent in chronic, but also allergic diseases [47,48,49,62,64]. Infection in the IPA patient could be a consequence of a single or limited exposure/inoculation event but the same cannot be said in CPA patients and other chronic diseases, e.g., cystic fibrosis (CF). How do we tell the difference between microevolution and succession, where different strains coexist with presumably equal potential for exacerbations? Microsatellite typing has shown microevolution of *A. fumigatus* in CPA patients with aspergillomas [48]. In this study, eight isogenic isolates displaying three different triazole resistance mechanisms were found within the same patient over the course of 1.5 years, suggesting the adaptability of *A. fumigatus* to drug therapy in the CPA setting [48]. Another single patient study analysed 92 *A. fumigatus* isolates obtained from aspergillomas of three CPA patients [49]. Although a level of microevolution was shown in two patients, the diversity in the third patient was clearly due to colonisation by multiple *A. fumigatus* genotypes. Arguably, microsatellite typing may not have had the resolution to determine whether the strains in these studies were identical, particularly as they showed major phenotypic and growth differences [48,49]. These two studies highlight the complexity of genotyping in CPA patients where the potential for colonisation by multiple stains complicates analyses due to the difficulty in assessing whether genotypes are genuinely isogenic unless NGS/WGS data are available. Similar findings in a study of CF patients suggest that numerous mechanisms explain the genetic landscape of *A. fumigatus* in chronically colonised patients [72]. This is further illustrated in a study of WGS data used to assess the genetic adaptability of thirteen *A. fumigatus* isolates obtained over a period of two years from a single patient with recurrent aspergillosis [64]. Microsatellite typing did not provide sufficient resolution in this study, but WGS demonstrated the existence of in-host microevolution by identifying a total of 248 SNPs, including a wide range of resistant SNPs, which were thought to have developed during the course of infection and as a result of antifungal therapy.

Clearly, assessing genotypes to determine the microevolution and/or succession of *A. fumigatus* in CPA patients is complicated by the reality of coinfection with multiple strains, as has also been shown in the CF lung [73] and reviewed in Vanhee et al. [27]. These studies showed the full spectrum of epidemiology, from unique genotypes in a significant proportion of CF patients to those with a predominant genotype or genotypes that succeeded each other, and patients with mixed coexisting genotypes. To dissect these relationships fully requires the discriminatory power of WGS/NGS performed over a significant period and abundance of contiguous sampling, requirements that are not easy to achieve.

### 4.3. Routes of Transmission

Patients can be colonised/infected with ubiquitous, airborne *A. fumigatus* spores anywhere at any time [74,75], attesting to the vigour of this species. Genotyping can disclose the source of infection, which facilitates the development of preventive measures, focusing on the living environment of vulnerable patients such as domiciles and hospitals. It can also reveal genotypic distinction between clinical isolates and environmental isolates and therefore exclude a common source during a suspected outbreak of nosocomial infection, usually associated with IPA, as reviewed recently [76,77]. Here, we present evidence pertinent to the IPA setting that may be relevant to CPA patients in the context of the increasing global risk of infection from environmental sources of *A. fumigatus* triazole-resistant strains.

Hospital-acquired aspergillosis is associated with environmental contamination, with genetic diversity reportedly high among indoor sampling sites, particularly during renovations [10,35,78,79,80,81,82,83,84,85,86]. For example, *Aspergillus* spp. can be isolated not only from the air and air conditioning but also from equipment and multiple hospital sites, such as carpets, walls, beds and blankets, sinks, trolleys, and medical devices [80]. Several patients attending particular clinics, e.g., the intensive and emergency care units in a Portuguese hospital, carried *A. fumigatus* strains with identical microsatellite genotypes associated with the one found in that unit, suggesting that these patients were colonised during their hospitalisation [40]. A nosocomial origin of *A. fumigatus* infections was also seen in lung transplant recipients, underlining the need for careful environmental monitoring of units in which high-risk patients are housed [87]. Genotyping studies have instructed prevention strategies for healthcare-associated aspergillosis transmission during hospital renovations [88]. Moreover, genotyping studies have informed recommendations for environmental infection control in healthcare facilities [89], particularly with respect to ventilation systems during renovations [90,91,92].

It has been shown that along with air as a major source for *A. fumigatus* contamination, water is also a source of strains causing infections [12,78]. In one hospital study in Norway, *A. fumigatus* strains recovered from locations inside and outside the hospital clustered in different genetic groups based on whether they were isolated from air or water. Moreover, there was close genetic relatedness between strains recovered from some IPA patients and air or water strains, attesting to the requirement for control measures to limit environmental hospital transmission [12]. In a fatal case of IPA, the STRA*f* genotypes of *A. fumigatus* strains isolated from a patient’s home (bedroom, bathroom, and basement) were found to be identical to the clinical isolate [13]. This case highlights the importance of environmental monitoring of the living environment of high-risk patients.

Case studies have suggested patient transmission is rare but possible [93,94], although less likely, between CPA patients. An interesting study described a hospitalised patient who was colonised by *A. fumigatus* during their hospital stay and became a source of airborne *A. fumigatus* contamination. Careful STRA*f* genotypic analysis of air and patient respiratory samples revealed not only the time frame during which the patient acquired their *A. fumigatus* strain but also that the patient was subsequently the source of *A. fumigatus* contamination in other rooms during their stay [93]. More evidence for potential patient-to-patient transmission was revealed by isogenic *A. fumigatus* STRA*f* genotypes from two CF patient cough samples in a recent study [95]. Notwithstanding the few case reports of patient transmission of *A. fumigatus*, environmental sources are most likely to be the main route of transmission.

### 4.4. Geographical Distribution of the Genetic Diversity of *Aspergillus fumigatus*

International and multicentre genotyping studies facilitate our understanding of the global genetic diversity and population structure of *A. fumigatus*. A recent review concluded that these techniques, particularly if used in isolation, did not show clustering among environmental and clinical isolates or any geographical clustering [32], which is generally substantiated in this update. In an early study using multilocus enzyme electrophoresis (MLEE), Bertout et al. revealed extensive genetic variability in 50 *A. fumigatus* isolates from 11 IPA patients obtained from three hospitals within a small geographic area, with significant diversity among geographic sites but less diversity within sites [96]. They recommended considering the local epidemiology of *A. fumigatus* for geographically distinct areas. In contrast, a later international study of environmental and clinical samples from Africa, Europe, North and South America, and Asia found no evidence to demonstrate a clear correlation between genotype and geography using a more discriminative microsatellite typing method [42]. Klaassen et al. used CSP typing to analyse their collection of 209 Dutch clinical isolates [34] and compared their results to North American genotyping data [35]. Both studies assigned the same most common CSP genotypes in their collections, suggesting a global distribution but a lack of clearly geographically separated *A. fumigatus* populations. However, a later study by the same group, using a more extensive set of genotyping markers including STRA*f*, CSP, and MLST typing, revealed evidence that Dutch clinical and environmental isolates were genetically differentiated into five distinct populations, suggesting a predominantly clonal mode of expansion but no geographic relationship [97]. Further, in a multicentre study in India, the distribution patterns of genotypes derived by combining PCR fingerprinting, MLST, STRA*f* microsatellite, and mating types varied widely among clinical and environmental strains, with some genotypes found in different locations while others found clustered together in the same geographical region, also suggesting clonal expansion of the latter population [98]. In a later study using CSP genotyping alone, clinical and environmental samples from different geographic regions were analysed, adding to the representation of the global *A. fumigatus* population [99]. Prevalent CSP genotypes were reported to be present in multiple countries; however, some types were found to be country-specific. Ultimately, this study found no distinctive population structures, although the greatest genetic diversity was found in Mexico and the least in French *A. fumigatus* populations. Finally, in an extensive genotyping study that assessed the progress made in dissecting the geographical genetic diversity of *A. fumigatus*, STRA*f* microsatellite markers were used to analyse 2026 *A. fumigatus* isolates from 13 countries in 4 continents [100]. Their evidence demonstrated a low, but significant, contribution of geographical isolation and adaptation to ecological niches to the diversity of *A. fumigatus* segregated among eight broad genetic clusters. Moreover, the study demonstrated significant evidence of gene flow, both between environmental and clinical isolates and between geographic populations, giving rise to a limited, but significant, genetic differentiation among geographic populations [100].

Notwithstanding the difficulties of establishing the relationship between genetic diversity and geographic distribution using different genotyping methods, the consensus is that the high genetic variability in the population structure and the broad distribution of *A. fumigatus* genotypes worldwide is not solely based on asexual reproduction as alluded to in previous studies [97,100,101,102]. Several genotyping studies incorporating mating type loci in their analyses demonstrated their resolving power within otherwise isogenic groups [12,69,98,103], although mating markers do not play a role in virulence per se [104]. The existence of a sexual cycle in *A. fumigatus* was only discovered recently and seems to occur more frequently than previously thought [101,102], as evidenced by the high genetic diversity of environmental isolates obtained from a compost heap, suggesting sexual or parasexual recombination events, rather than clonal reproduction [105]. Confirmation of this theory was provided in a laboratory study in which the sexual cross between two TR_46_ isolates from the same triazole-containing compost resulted in the recovery of a novel pan-triazole-resistant mutation (TR_46_ (3)/Y121F/M172I/T289A/G448S) [106]. However, different geographic substructures were found among triazole-resistant and susceptible populations, with resistance to multiple antifungal triazole drugs being driven by clonal expansion rather than sexual recombination [100]. Clearly, the incorporation of resistance markers in genotyping investigations provides further epidemiological resolution.

## 5. Genetic Diversity and Geographical Distribution of Triazole-Resistant A. fumigatus Strains

Triazole resistance complicates the diagnosis and treatment of aspergillosis and the resulting therapeutic failures significantly increase mortality rates [4,107]. The global prevalence of triazole-resistant *A. fumigatus* is a major clinical concern and therefore guidelines on the management and diagnosis of *Aspergillus* diseases, including CPA, recommend resistance testing in combination with local resistance surveillance [3,108].

Two routes of antifungal resistance acquisition are identified, both due to selective pressures from the extensive use of antifungals [109,110]. Long-term triazole antifungal drug treatment can facilitate selection of *A. fumigatus* resistance by causing in-host microevolution [111,112]; these mechanisms are mainly due to nonsynonymous mutations in the lanosterol 14α-demethylase drug target, *cyp51A*, and have been reviewed elsewhere [4,113,114]. Unusually, in a recent case study of fatal aspergillosis, STR*Af* typing and WGS revealed the acquisition of a new triazole resistance mechanism (TR_120_) in vivo due to long-term triazole therapy [115]. The second identified route is through the dissemination of triazole-resistant isolates in the environment due to extensive use of agricultural azole fungicides (C14-demethylase inhibitors (DMIs)) related to those used clinically [116]. These mutations are characterised by specific *cyp51A* SNPs coincident with tandem repeat sequence insertions upstream of the gene, abbreviated as TR34/L98H, known to confer pan-azole resistance, and TR46/Y121F/T289A, which confers voriconazole resistance [109]. Evidence for environmental transmission is shown by the fact that patients with no known history of clinical triazole exposure harbour triazole-resistant strains [117,118,119], increasingly supported by microsatellite and CSP genotypic data [120,121] and reviewed recently [122,123]. An interesting study supporting the development of *A. fumigatus* environmental TR46/Y121F/T289A resistance due to fungicide exposure in compost heaps found that the prevalence of triazole-resistant isolates from the compost with triazole exposure was 91%, and only 2% in the compost without triazole exposure [106]. Another Dutch study determined environmental plant waste hot spots and reservoirs for the development of both TR34/L98H and TR46/Y121F/T289A resistance along with guidance on preventative measures [124]. The scientific literature has seen an explosion of accounts of *A. fumigatus* triazole resistance of putative environmental origin in clinical isolates in the last decade [4,113,125,126,127]. However, the debates about whether the origin of some resistance mechanisms is purely environmental in origin [126] or due to agricultural fungicide application [128] or not [129] continue and could be clarified if genotyping of all strains was routinely included in such studies. Since patient-to-patient transmission is possible but rare, it is unlikely that long-term triazole therapy in patients has a wider effect on the community. Therefore, environmental sources are likely to represent a greater and more continuous burden of triazole-resistant *A. fumigatus* strains.

Genotyping data from the Netherlands and various other European countries demonstrated reduced genetic diversity of TR34/L98H isolates compared to those with other resistance mechanisms or wild-type control strains, suggesting a common ancestor for and recent emergence of this resistance mutation [50]. It is suggested that the TR34/L98H resistance mutation would have emerged around 1997 in the Netherlands and migrated across Europe where, since then, genetically related TR34/L98H isolates have been found in France, Germany, Italy, Spain, and the United Kingdom [46,51,63,118]. However, the migration of TR34/L98H strains is not limited to Europe, as genotypic analyses of Japanese strains revealed clustering with Dutch and French strains [52]. Environmental *A. fumigatus* isolates in Iran have also been found to display a single TR34/L98H genotype closely related to Dutch and Indian isolates [53].

Interestingly, microsatellite genotypic analysis has revealed local clusters in India not closely related to triazole-resistant TR34/L98H isolates from the Netherlands and other European countries, first reported in 2012 [54]. Microsatellite genotypic analysis revealed a unique genotype across all Indian triazole-resistant clinical and environmental isolates tested, distinct from Chinese, Middle Eastern, and European TR34/L98H strains [55,56]. It has been suggested that this distinctive genotype was derived from a cross between a triazole-resistant strain migrated from outside of India and a native triazole-susceptible strain from within India followed by recombination [55]. Recent reports showing genetic relatedness among isolates from India, Kuwait, and Iran suggest that Indian triazole-resistant isolates have migrated outside Asia [57,58].

The phenomenon of localised genetic TR34/L98H clusters has been recorded in other countries. It is thought that Chinese TR34/L98H/S297T/F495I strains developed under the selection pressure of imidazole fungicides [59]. In another study in China, both unique and clonal genotypes were found among clinical isolates harbouring TR34/L98H resistance profiles [130]. In Germany, local factors were suggested to cause the increased prevalence of specific genotypes not found elsewhere, although there was evidence of dispersion of known genotypes from the Netherlands [51]. Cameroonian strains demonstrated significantly novel allelic and genotypic diversity compared to *A. fumigatus* populations reported in Europe, Asia, and North America [60]. In Taiwan, both the global spread of TR34/L98H isolates and local genetic clustering of clinical and environmental TR34/L98H has been observed [61], although local clustering of microsatellite genotypes was not limited to TR-related resistance strains. In Romania, as well as in India, unique microsatellite profiles were also found among the environmental triazole-resistant isolates harbouring the G45E mutation [131].

Several broad analyses incorporating genotyping of global *A. fumigatus* strains have revealed insights into the mechanisms of acquisition of environmental triazole resistance, confirming the role of sexual recombination events as a basis for diversification and localised clonal expansion once triazole-resistant strains have adapted and become established [14,100,123]. Review of this evidence in a timely manner reveals progressive developments in our understanding of these mechanisms and relies on the more discriminative genotyping methods. The earliest WGS study showed that the genetic diversity of twenty-four triazole-resistant *A. fumigatus* strains was equivalent within countries and between continents, except for India, where the recent clonal expansion was explained by a highly adapted genotype [63]. This study demonstrated that there was no phylogenetic difference between clinical and environmental strains and that azole resistance due to TR34/L98H could occur in diverse genetic backgrounds, in both clinical and environmental isolates [63]. A later study of a considerably larger strain set reported low but statistically significant genetic diversity among geographic and ecological populations of *A. fumigatus* using STRA*f* genotyping [100]. Further, this study did demonstrate differences among triazole-resistant and susceptible strains, commensurate with adaptation to localised drug pressures. Finally, an extensive STR*Af* study using 4049 *A. fumigatus* worldwide strains revealed that *A. fumigatus* was subdivided into two broad clades [43], with the environmental mutations TR34/L98H and TR46/Y121F/T289A unevenly distributed across them. Identical STR*Af* genotypes were isolated from both clinical strains and environmental locations and found globally distributed, confirming triazole resistance as an international public health concern. However, the genetic differences demonstrated in the previous large dataset study [100] were confirmed, demonstrating that resistant strains were significantly less diverse than triazole-susceptible ones [43].

Clearly, there are concerns about the geographical distribution of *A. fumigatus* resistant genotypes. Natural forces such as wind may contribute to the migration of the airborne *A. fumigatus* conidia as well as the impact of human travel and trade. Identical genotypes have been found across multiple centres and countries, sometimes separated by thousands of kilometres [55]. This theory was recently confirmed in New Zealand, one of the most isolated countries, which should have genetically unique populations if migration were non-existent. STR*Af* analysis of 104 Auckland soil samples showed mixed origins, including both indigenous as well as recently introduced genetic elements from other geographic areas [132], reinforcing the role for microsatellite genotypic analysis and continual local surveillance.

## 6. Genotyping of *Aspergillus fumigatus* Isolates from the UK National Aspergillosis Centre

The importance of local resistance surveillance, along with a paucity of CPA genotyping studies, suggested closer monitoring of the patients attending the UK National Aspergillosis Centre (NAC) clinic. We performed a small study using our database of clinical *A. fumigatus* isolates derived from CPA patients located in different geographical regions of the UK, who had visited the clinic for more than 2 years [133]. A total of 31 *A. fumigatus* isolates collected from 14 CPA patients, along with two reference strains, were analysed by both nSTR*Af* and TRESPERG genotyping methods [40,46].

**Figure 1:**
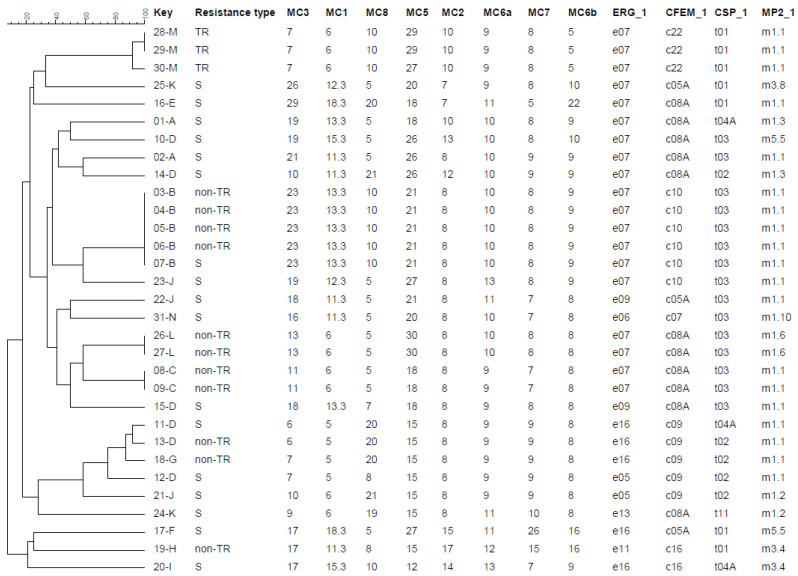
Dendrogram of 29 clinical chronic pulmonary aspergillosis (CPA) and 2 reference isolates. MC3-MC6b were the 8 markers of the nSTR*Af* method [40]. ERG_1-MP2_1 were the 4 markers of the TRESPERG method [55]. TR represents tandem repeat in the *cyp51A* gene and non-TR represents resistance types other than tandem repeat, while S represents triazole-sensitive (itraconazole, voriconazole, posaconazole, and isavuconazole were tested using the EUCAST method [134]). More than three isolates per patient were analysed in four of fourteen patients, designated by unique capital letters.

A total of 23, 20, and 24 genotypes were identified by nSTR*Af*, TRESPERG, and the two methods combined, respectively, indicating significant diversity (Figure 1). All identical genotypes were found in strains collected from the same patients. In contrast, several different genotypes were found in some patients (A, D, J, K), suggesting multi-colonisation by *A. fumigatus*. Identical genotypes with different triazole resistance patterns were also observed (patients B and D), which suggested resistance occurred due to long-term treatment with antifungal drugs. On the other hand, strains with identical susceptibilities that differed in the repeat numbers in several markers were also found, possibly explained by microevolution.

**Figure 2:**
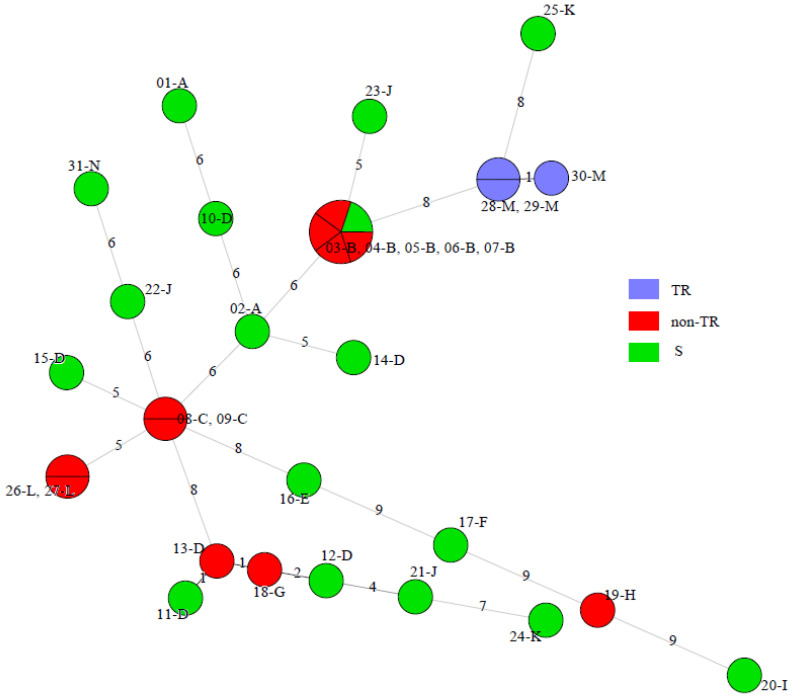
Minimum spanning tree of 29 clinical and 2 reference *A. fumigatus* isolates generated by considering the combined nSTRA*f* and TRESPERG data. Each circle shows a unique genotype. The different colours of the circles indicate the *cyp51A* modifications, grouped as triazole-susceptible (S), triazole-resistant with tandem repeats (TR), and triazole-resistant with nonsynonymous *cyp51A* mutations (non-TR).

As expected, isolates harbouring a tandem repeat (TR) resistance mechanism grouped together in one cluster as they originated from one patient, while strains with triazole susceptibility (S) or triazole resistance due to nonsynonymous *cyp51A* mutations (non-TR) were distributed in several clusters (Figure 2). Although limited, these data suggest that non-TR strains are as genetically diverse as susceptible isolates in this snapshot population and more diverse than TR triazole-resistant isolates.

The combined nSTRA*f* and TRESPERG method seemed to have sufficient discriminatory power for epidemiology investigation, only limited due to the small number of patients and lack of environmental isolates. However, what is demonstrated by these data is the importance of considering non-TR resistance alongside genotyping data to reveal not only the genetic diversity of *A. fumigatus* over time, but also the origin and evolution of resistance over extended periods of antifungal treatment. This may inform the timeframe for the development of resistance in the CPA setting and could eventually lead to better treatment outcomes for CPA patients.

## 7. Conclusions

Alongside the usual consequences of molecular epidemiological studies informing environmental monitoring and clinical surveillance, genotyping data combined with resistance and mating type markers have allowed for the elucidation of the mechanisms for the global diversity of *A. fumigatus*. This has been accomplished by the significant improvements to genotyping techniques over the last 20 years and the realisation of the minimal criteria by which diversity can be detected and monitored. The dawn of more widely available NGS/WGS techniques for elucidating the genetic diversity of *A. fumigatus* should address the paucity of epidemiological studies of chronic aspergillosis conditions and decipher the mechanisms of resistance development in this more complicated setting. However, a significant limitation of genotyping studies to date is their reliance on culture. Having introduced the use of NGS/WGS methods, the next major advance is to engage this technology and shed light on the actual diversity in the lungs using patient respiratory samples directly. The current knowledge about diversity and resistance profiling may be turned on its head once mycobiomes from different patient groups are revealed and representatives of different sources are compared globally using more diverse loci than are currently considered.

## Figures and Tables

**Table 1:** Overview of *A. fumigatus* genotyping approaches and their features.

Typing Technique	Principle	Discriminatory Power ^1^	Advantage(s)	Disadvantage(s)	Reference(s)
TRESP	hypervariable TRs ^2^ in 3 genes	>0.997	specialist equipment not required ease of interpretation, reproducibility	lower resolution	[44]
	including *cyp51A* resistance markers	0.890			
TRESPERG	hypervariable TRs in 4 genes	>0.997			[46]
	including *cyp51A* resistance markers	>0.993			
TRESPERG + STRA*f*	hypervariable TRs in 4 genes plus 9 STRs ^3^ and *cyp51A* resistance markers	>0.999	high-resolution fingerprinting	technical expertise specialist equipment	[46]
STRA*f*	9 STRs	>0.999	high-resolution fingerprinting	technical expertise specialist equipment reproducibility interlaboratory variation	[30]
nSTRA*f*	8 STRs	0.9997	high-resolution fingerprinting single multiplex reaction longer, more accurate STRs	technical expertise specialist equipment	[40]
STRA*f*+	9 STRs and *cyp51A* resistance markers	na ^4^	high-resolution fingerprinting public database (AfumID)	technical expertise specialist equipment	[43]
NGS/WGS ^5^	genome	>0.999	maximum resolution	technical expertise specialist equipment bioinformatics capabilities	[65,66]

^1^ Based on Simpson’s diversity index. ^2^ TR, tandem repeat. ^3^ STR, short tandem repeat polymorphism microsatellite markers; also referred to as microsatellite length polymorphisms (MLP) or variable number of tandem repeats (VNTR). ^4^ na, not available. ^5^ NGS, next-generation sequencing; WGS, whole-genome sequencing.

## Data Availability

The data presented in this study are available on request from the corresponding author. The data are not publicly available due to the work being part of a University of Manchester Master in Science dissertation.
